# Comparison of clinicopathological and genomic profiles in anal squamous cell carcinoma between Japanese and Caucasian cohorts

**DOI:** 10.1038/s41598-023-30624-w

**Published:** 2023-03-03

**Authors:** Takahiko Ito, Daisuke Takayanagi, Shigeki Sekine, Taiki Hashimoto, Yoko Shimada, Maiko Matsuda, Masayoshi Yamada, Ryuji Hamamoto, Tomoyasu Kato, Dai Shida, Yukihide Kanemitsu, Narikazu Boku, Takashi Kohno, Atsuo Takashima, Kouya Shiraishi

**Affiliations:** 1 Department of Gastrointestinal Medical Oncology, National Cancer Center Hospital, Tsukiji 5-1-1, Chuo-ku, Tokyo 104-0045 Japan; 2 Department of Gastroenterology and Hepatology, Graduate School of Medicine, Kyoto University, 54 Shogoin-kawahara-cho, Sakyo-ku, Kyoto 606-8507 Japan; 3 Division of Genome Biology, National Cancer Center Research Institute, 5-1-1 Tsukiji, Chuo-ku, Tokyo 104-0045 Japan; 4 Department of Clinical Pathology, National Cancer Center Hospital, Tsukiji, Chuo-ku, Tokyo 104-0045 Japan; 5 Endoscopy Division, National Cancer Center Hospital, Tsukiji, Chuo-ku, Tokyo 104-0045 Japan; 6 Division of Molecular Modification and Cancer Biology, National Cancer Center Research Institute, Tsukiji, Chuo-ku, Tokyo 104-0045 Japan; 7 RIKEN Center for Advanced Intelligence Project, Cancer Translational Research Team, Hirosawa, Wako, Saitama 351-0198 Japan; 8 Department of Gynecology, National Cancer Center Hospital, Tsukiji, Chuo-ku, Tokyo 104-0045 Japan; 9 Department of Colorectal Surgery, National Cancer Center Hospital, Tsukiji, Chuo-ku, Tokyo 104-0045 Japan; 10 Division of Frontier Surgery, The Institute of Medical Science, The University of Tokyo, Shirokanedai, Minato-ku, Tokyo 108-8639 Japan

**Keywords:** Cancer genetics, Anal cancer

## Abstract

Anal squamous cell carcinoma (ASCC) is a rare tumor of the gastrointestinal tract. We aimed to compare the genetic backgrounds and their effect on clinical outcomes between Japanese and Caucasian patients with ASCC. Forty-one patients diagnosed with ASCC at the National Cancer Center Hospital were enrolled and evaluated for clinicopathological features, human papillomavirus (HPV) infection, HPV genotypes, p16 expression, PD-L1, and association of p16 status with the efficacy of concurrent chemoradiotherapy (CCRT). Target sequencing for hotspot mutations in 50 cancer-related genes was performed using genomic DNA from 30 available samples. Of 41 patients, 34 were HPV-positive (among them, HPV 16 was predominant; 73.2%); 38 patients were p16-positive (92.7%); and 39 patients received CCRT, of whom 36 were p16-positive and three p16-negative. p16-positive patients showed better complete response than p16-negative patients. Among 28 samples, 15 showed mutations in *PIK3CA, FBXW7, ABL1, TP53,* and *PTEN*; no difference in mutation profiles between the Japanese and Caucasian cohorts was observed. Actionable mutations were detected in both Japanese and Caucasian patients with ASCC. Genetic backgrounds, such as the HPV 16 genotype and *PIK3CA* mutations, were common regardless of ethnicity. p16 status may be a prognostic biomarker for CCRT in Japanese patients with ASCC.

## Introduction

Anal cancer is an uncommon disease, which occurs in the anal canal and is divided mainly into two histological types: adenocarcinoma and squamous cell carcinoma^1^. The standard treatment for adenocarcinoma of the anal canal is similar to that for low rectal cancer, that is, radical surgery with/without perioperative chemoradiation therapy. In contrast, anal squamous cell carcinoma (ASCC) is sensitive to radiation. As the treatment strategy for anal cancer differs depending on the histological type, the clinical issues need to be analyzed separately. In 2008, the worldwide incidence of ASCC was reported as approximately 27,000 new cases per year^2^, and it has slightly increased in developed countries over the past few decades^2–6^.

ASCC commonly arises near the squamocolumnar junction and is considered to be caused by human papillomavirus (HPV) infection^7^. Most ASCC cases are HPV-positive, especially HPV 16, which is a high-risk HPV, confirming previously reported genotypes^7–10^. However, some ASCC cases are non-HPV 16/18^7^. Other previously reported risk factors for the development of ASCC include pharmacological immune suppression as in the case of solid organ transplant recipients, immunosuppression related to human immunodeficiency virus (HIV)^8^, smoking^11^, and sexually transmitted infections^2^. However, these previous studies reported data from western countries, with fewer reports from Asia. In addition, because few studies have reported HPV genotyping for Japanese or Asian patients with ASCC, there is a need to elucidate the difference in the distribution of HPV genotypes between Caucasian and Asian populations.

The global standard treatment for advanced ASCC without distant metastasis is concurrent chemoradiation therapy (CCRT). According to the ACTII trial, three-year locoregional control and overall survival (OS) of patients with ASCC were 74% and 82%, respectively^12^. In Japan, five-year progression-free survival (PFS) and OS of patients with stage II/III ASCC were reported to be 85.7% and 87.3%, respectively^7^. As reported in these clinical trials, the outcome of ASCC without distant metastatic lesions treated with CCRT is relatively good, but some cases are refractory, and their prognosis is poor. Therefore, it is necessary to clarify the genetic and/or immunological backgrounds of patients with ASCC refractory to CCRT for the development of new treatments, such as molecular targeted therapies and immune checkpoint inhibitors. In a meta-analysis, HPV infection, p16^INK4a^ (p16) overexpression, and wild-type p53 were defined as predictive markers for CCRT and prognostic factor^13–15^; however, these previous studies reported the data of patients in western countries and the association between these predictive markers and clinical outcomes in Asian patients with ASCC remains unclear.

To properly apply molecular targeted therapies, the distribution of mutation patterns linked to molecularly targeted drugs needs to be identified in each cancer type. In western countries, many studies have focused on somatic mutational analysis for ASCC^10,16^, and the most frequently detected genomic mutation is the *PIK3CA* mutation, with a frequency of 30%. *PIK3CA* mutations are frequently detected in many cancers, including breast, lung, and colon cancers^17^. The PI3Kα-specific inhibitor, alpelisib, has shown antitumor activity in human epidermal growth factor receptor 2 (HER2)-negative breast cancer with *PIK3CA* mutation^18^. However, few studies report somatic genotyping for Japanese or Asian patients with ASCC.

Immune checkpoint inhibitors serve as the standard treatment for refractory ASCC, and anti-programmed cell death protein-1 (PD-1) therapies have emerged for various cancers, including ASCC. Furthermore, programmed death ligand-1 (PD-L1) expression rate is used as a predictive factor^19,20^. Elucidation of the frequency of PD-L1 expression in Asian patients with ASCC is important.

In this study, we compared the genetic backgrounds, such as HPV infection, HPV genotypes, p16 expression, PD-L1 expression, and other mutational patterns, and their effect on clinical outcomes after CCRT between Japanese and Caucasian patients with ASCC.

## Results

### Patient characteristics

A total of 41 patients were selected for this study, and their characteristics are summarized in Supplementary Table S1. The median follow-up time for all patients was 64.3 months (13.5–162.3). The median age was 65.0 (range, 29–82 years), and 32 patients (78.0%) were women. One (2.4%), 20 (48.8%), 18 (43.9%), and 2 (4.8%) patients had I, II, III, and IV ASCC, respectively. All patients were negative for HIV infection. For treatment, 39 patients (95.1%) had received concurrent chemoradiotherapy (CCRT) and one patient had received radiotherapy alone, and the best supportive care was selected for the remaining one patient owing to poor medical condition.

Of the 39 patients treated with CCRT, 34 had received fluorouracil plus mitomycin C and five had received fluorouracil plus cisplatin. The median radiotherapy dose was 54.9 Gy (50.4–70 Gy). One case was excluded from the analysis because the image could not be evaluated. Thirty-one patients (81.6%) achieved complete response (CR) (Table 1). Progressive disease was observed in six patients (15.8%) (Table 1).

**Table 1:** Comparison of clinicopathological factors and clinical outcomes according to p16 status.

Variable	p16 IHC		*p* Value
	Positive (n = 38)	Negative (n = 3)	
Age (year), Median (range)	65.0 (29–82)	71.0 (40–73)	0.78**
*Sex, n (%)*			0.11*
Female	31 (81.6)	1 (33.3)	
Male	7 (18.4)	2 (66.7)	
*Clinical Stage, n (%)*			0.91*
I	1 (2.6)	0 (0.0)	
II	18 (47.4)	2 (66.7)	
III	17 (44.7)	1 (33.3)	
IV	2 (5.3)	0 (0.0)	
*Expression of PD-L1, n (%)*			0.23*
Positive (≧1%)	19 (50.0)	0 (0.0)	
Negative (< 1%)	19 (50.0)	3 (100)	
*HPV genotype*			0.0077*
HPV 16	30 (79.0)	0 (0.0)	
HPV 18	1 (2.6)	0 (0.0)	
Other HPV genotypes	3 (7.9)	0 (0.0)	
Negative	4 (10.5)	3 (100)	
*Best overall response of CCRT, n (%)*	n = 35	n = 3	
Complete response	30 (85.6)	1 (33.3)	0.08*
Partial response	3 (8.6)	1 (33.3)	
Stable disease	1 (2.9)	1 (33.3)	
Progressive disease	1 (2.9)	0 (0.0)	

*CCRT* Concurrent chemoradiation therapy.

*Fisher's exact test, **Mann–Whitney U test.

### HPV infection, HPV genotypes, and p16 expression in ASCC tissues

Of the 41 patients, 34 ASCC tumor samples (82.9%) were HPV-positive, and the most frequent HPV genotype was HPV 16 (73.1%) (Supplementary Table S2). HPV 18, HPV 33, HPV 35, and HPV 58 were detected in one patient (2.4%) each. p16 positivity was found in 38 patients (92.7%) (Supplementary Fig. S1)^21^, and 34 of the 38 patients (89.5%) showed HPV infection (Table 1). HPV was not detected in the remaining three patients without p16 expression.

### Frequency of genomic alterations and actionable mutations in 30 patients with ASCC

**Figure 1:**
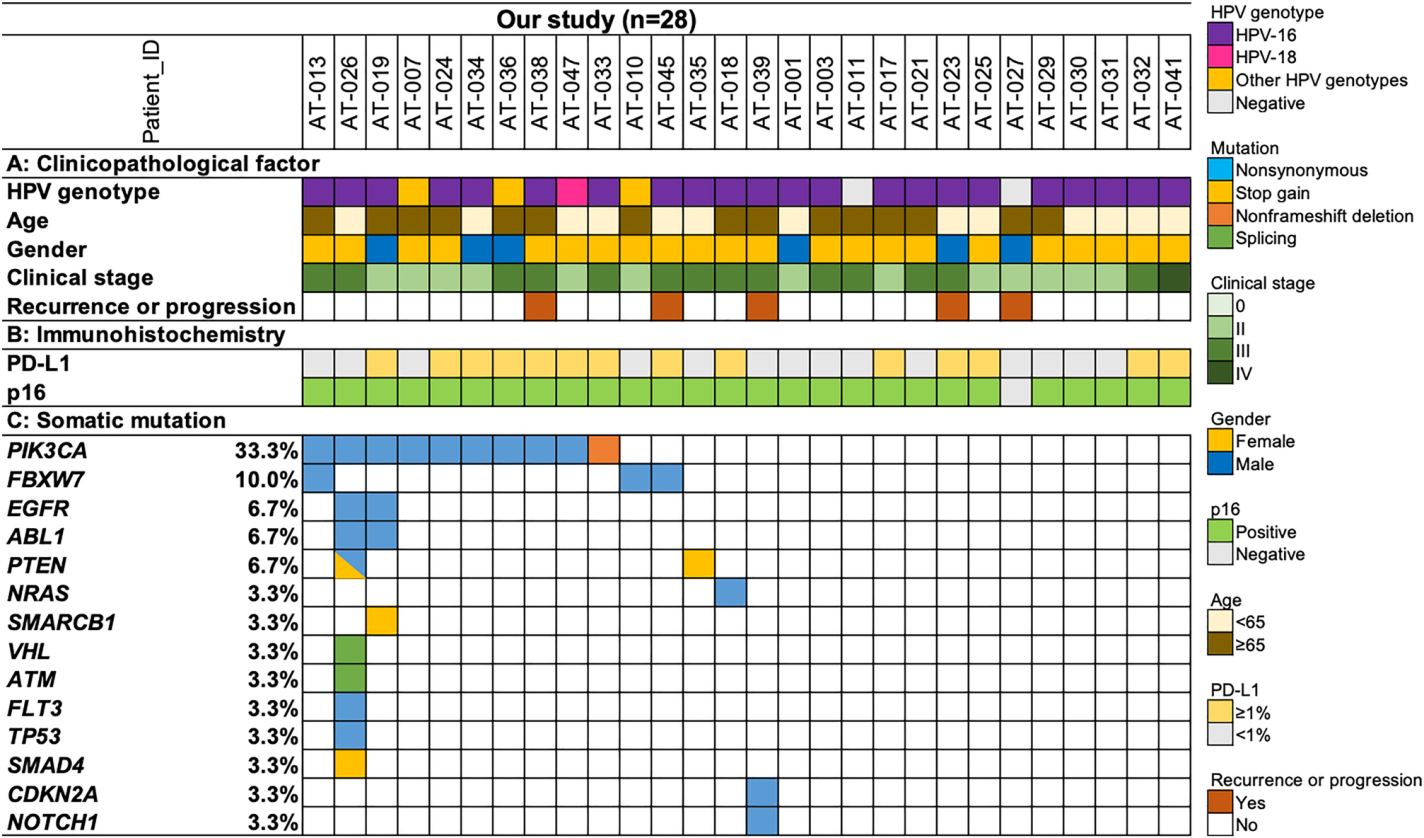
Profile of 28 Japanese patients with ASCC. All panels are aligned with vertical tracks representing 28 patients with ASCC. (**a**) The pattern of clinicopathological factors. (**b**) Evaluation of PD-L1 and p16 expression using IHC. (**c**) Distribution of somatic mutations detected in Japanese patients with ASCC. ASCC, Anal squamous cell carcinoma; IHC, Immunohistochemistry.

Of the 41 patients, high-quality sequencing data could be obtained from 28, using DNA extracted from FFPE specimens. We identified 30 oncogenic/likely oncogenic or pathogenic/likely pathogenic mutations in the TCGA dataset or ClinVar database, respectively. These somatic mutations included 22 nonsynonymous, five stop-gain, one non-frameshift, and two 5′ untranslated region (UTR) mutations. The most frequently mutated gene, *PIK3CA,* was detected in ten (33.3%) patients, followed by *FBXW7, EGFR*, *ABL1,* and *PTEN* (Fig. 1, Supplementary Table S3). Actionable genomic alterations that had a level of evidence 1–3B in OncoKB were detected in 11 (36.7%) patients (Supplementary Fig. S3, Supplementary Table S4).

### Frequency of somatic and actionable mutations in GENIE

**Figure 2:**
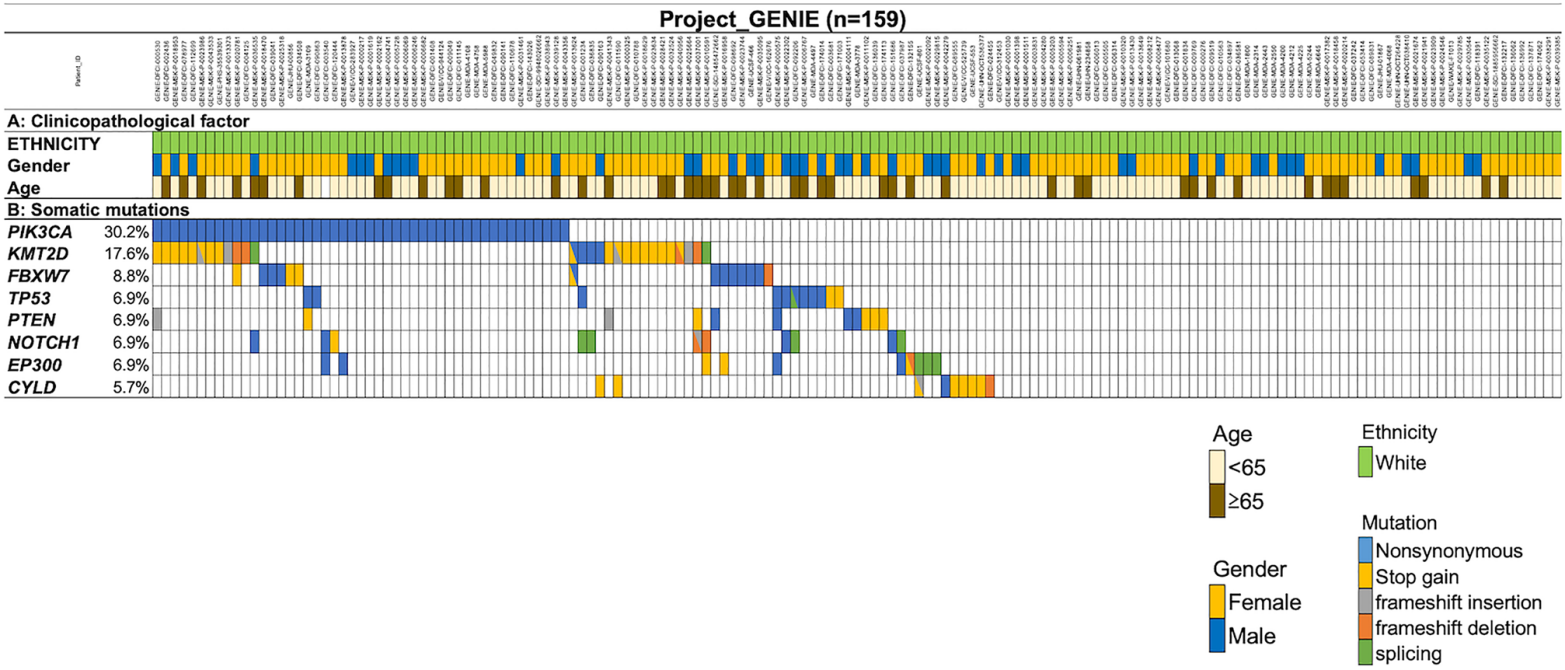
Profile of genomic alterations of patients with ASCC registered in GENIE. All panels are aligned with vertical tracks representing 159 individuals. (**a**) Patient characteristics. (**b**) Distribution of somatic mutations of patients with ASCC in GENIE. ASCC, Anal squamous cell carcinoma.

We analyzed the GENIE datasets of 159 Caucasian patients with ASCC^22^. The only available clinical characteristics were sex and age (Fig. 2). The most commonly mutated gene was *PIK3CA* (30.2%), followed by *FBXW7* (8.8%), *TP53* (6.9%), and *PTEN* (6.9%) (Fig. 2, Supplementary Table S5). There was no difference in the somatic mutation frequency between our cohort and GENIE data (Supplementary Table S6). Mutations on *KMT2D* (17.6%), *NOTCH1* (6.9%), *EP300* (6.9%), and *CYLD* (5.7%) were not evaluated in our study because these genes were not included in the Ion AmpliSeq Cancer Hotspot Panel v2. Actionable genomic alterations that were registered as the level of evidence 1–3B in OncoKB were detected in 56/119 (47.1%) patients (Supplementary Table S4).

### Efficacy of CCRT according to p16 status

**Figure 3:**
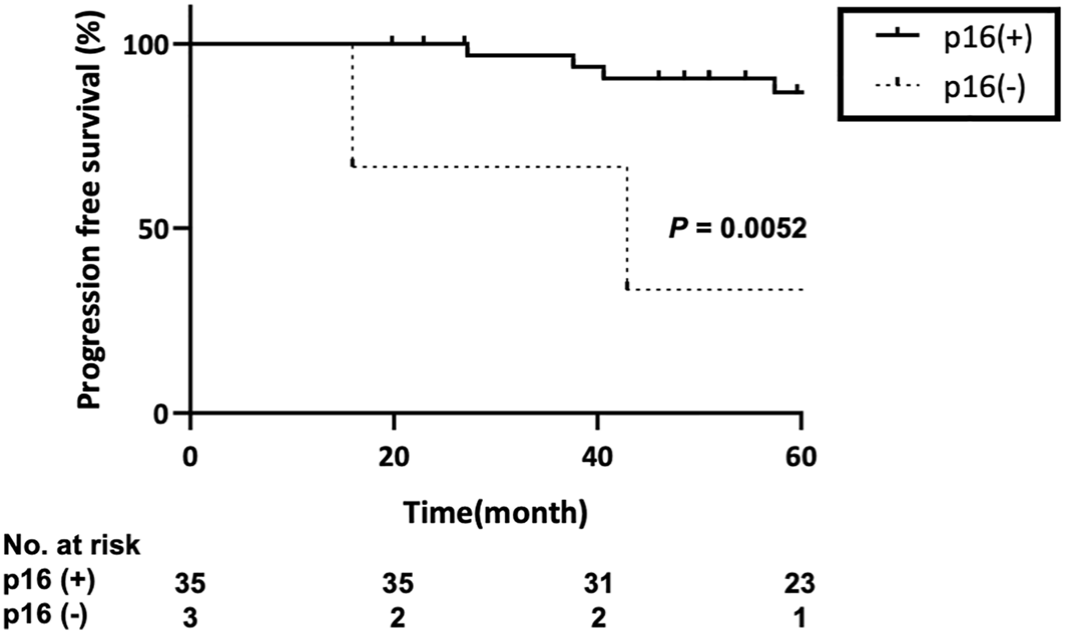
Kaplan–Meier analysis of p16 expression and progression-free survival in 38 patients receiving CCRT. Patients with p16 overexpression are indicated with a straight line and those without p16 expression with a dashed line. CCRT, Concurrent chemoradiation therapy.

Thirty-nine patients, 36 of whom were p16-positive and 3 were p16-negative, had received CCRT. Although 30 patients (83.3%) achieved CR in the p16-positive group, only one patient (33.3%) achieved CR in the p16-negative group (Supplementary Table S7). No significant difference between p16 expression and CCRT effect by stage was observed (Supplementary Table S8). Moreover, the PFS of the p16-positive group was significantly longer than that of the p16-negative group (*p* = 0.0052, Fig. 3). In contrast, there were no statistically significant associations between HPV genotypes and the clinical outcome (Supplementary Table S9).

### Expression of PD-L1 in ASCC tissues

Of the 41 patients with ASCC, 19 (46.3%) exhibited a tumor proportion score (TPS) of > 1% (Supplementary Table S8). All PD-L1-positive cases were p16-positive, and those with p16-negative tumors were PD-L1-negative. In addition, there was no association between PD-L1 expression and the clinical outcome (Supplementary Fig. S4).

## Discussion

This is the first study focusing on the comparison of clinicopathological features and genomic profiles in ASCC between Japanese and Caucasian cohorts. Of the 41 patients, 34 (82.9%) had the following HPV infections: HPV 16 (73.1%), HPV 18 (2.4%), HPV 33 (2.4%), HPV 35 (2.4%), and HPV 58 (2.4%). Our results suggested that the frequency and diversity of HPV did not differ between Japanese and Caucasian patients with ASCC. Furthermore, the distribution of HPV genotypes was similar to that in Japanese patients with cervical cancer^23,24^. HPV vaccination is important, regardless of ethnicity, to prevent the development of not only cervical cancer but also ASCC. In our cohort, Asian-specific genotypes, such as HPV 58, were detected; therefore, the development of HPV vaccines for specific HPV genotypes identified in Asians might also be useful.

In this study, somatic mutation analysis revealed that the most frequently mutated gene was *PIK3CA*. Several mutations were reported in ASCC, and the most common *PIK3CA* mutations were E542K (c.1624 G > A) and E545K (c.1633 G > A), detected both in our cohort and GENIE. The frequency and profiles of somatic mutations were similar between Japanese and Caucasian patients with ASCC. In addition, *PIK3CA* mutations are actionable mutations, which have been approved as predictive biomarkers for the use of the PI3K inhibitor alpelisib by the US Food and Drug Administration^18^. Furthermore, *NRAS* and *ATM* mutations detected in our cohort can be predictive biomarkers for molecularly targeted drugs, such as MEK and PARP inhibitors. Because molecularly targeted drugs act on a specific molecule that plays an important role in cancer proliferation and survival, new drug development based on these somatic mutations is expected.p16 is one of the most studied biomarkers in lower anogenital tract squamous intraepithelial lesions^25^ and is overexpressed frequently (> 80%) in patients with ASCC. p16 overexpression in ASCC is correlated with HPV positivity^26^. In contrast, lack of p16 expression in ASCC was found to be associated with a worse prognosis than p16 overexpression in a meta-analysis^27^. In our cohort, three cases without p16 expression were negative for HPV infection, which was in line with the previous studies reporting an association between HPV genotypes and p16 status^28^. In addition, it is well known that p16 is a predictive factor for CCRT in other types of cancers, such as oropharyngeal cancer^29^. Our data suggested that p16 status in ASCC could be a prognostic biomarker for CCRT in Asian countries, supporting previous studies conducted in western countries^21,30^. However, owing to the small number of p16-negative cases, further studies are needed in Asian patients with ASCC. Moreover, how the lack of p16 expression is associated with poor clinical outcomes after CCRT remains unclear, and its functional significance needs to be clarified in the future.

In this study, 19 (46.3%) patients showed PD-L1 expression with a TPS of > 1%. Previous studies showed that PD-L1 positivity is 63–74%, which is slightly higher than that observed in our study. The frequency of PD-L1 expression (TPS > 1%) in solid tumors varies in different tumors, i.e., non-small cell lung cancer (59.7%), endocrine tumors (47.1%), head and neck tumors (25.9%), and neuroendocrine tumors (15.4%)^31^. PD-L1 expression may be an important biomarker of the efficacy of anti-PD-1 treatments for patients with ASCC, and an anti-PD-L1 antibody may have the potential to serve as a treatment option not only for Caucasian patients but also for Japanese patients with ASCC.

This study has a few limitations that need to be addressed. First, the number of patients with ASCC included in this study was limited, and a significant difference could not be demonstrated; thus, further validation is warranted. However, considering the rarity of the disease, the mutational analysis of 28 cases with ASCC has been reported for the first time in Asia. Second, targeted sequencing for mutational analysis used in this study contained only hotspot mutations in 50 cancer-related genes. Nevertheless, we could identify therapeutic target candidates for ASCC, such as alterations in *PIK3CA* and PD-L1 expression, even for limited genes and their regions.

## Conclusions

We observed similar clinicopathological features and mutational patterns between Japanese and Caucasian patients with ASCC. Although our data have scope for enhancement, the results suggest that the lack of p16 expression in ASCC may be a poor prognostic biomarker for CCRT. However, we lack sufficient data in this regard. The frequency of PD-L1 expression in ASCC was higher than that in other solid tumors. Half of the patients with ASCC may have a chance to receive molecular targeted therapy.

## Materials and methods

### Clinical profiles of patients

This was a single-center, retrospective study consisting of consecutive case series. Forty-one patients diagnosed with ASCC between 2006 and 2018 at the National Cancer Center Hospital (NCCH) were identified for this study. The clinical stage was evaluated according to the Union for International Cancer Control (UICC) TNM 8th edition^32^. Clinical data, such as disease stage, age, sex, treatment modality, and therapeutic effects, were retrospectively collected from the existing medical records.

### Ethics approval and consent to participate

The general requirements for informed consent for the use of patient samples in the research were obtained at their first visit to the NCCH. Information obtained in this study using samples collected after obtaining general informed consent from participants has been summarized on the NCCH website (https://www.ncc.go.jp/jp/ncch/division/clinical_trial/info/clinical_trial/index.html). Patients were free to revoke their presumed consent at any time point. We only used samples from patients who did not revoke their consent. Patients who withdrew consent for the use of their residual samples were excluded from this study. This study protocol followed the ethical guidelines of the Helsinki Declaration and was approved by the Institutional Review Board of the National Cancer Center (2018-267). The data used in this study were anonymized before its use.

### Identification of HPV genotypes and detection of HPV infections

Genomic DNA was extracted from the cored formalin-fixed paraffin-embedded (FFPE) tissue samples using the QIAamp DNA FFPE Tissue Kit (Qiagen, Hilden, Germany) as per the manufacturer’s protocol. DNA (10 ng) was amplified via PCR, using TaKaRa Taq DNA polymerase (Takara Bio Inc., Shiga, Japan) and a pair of consensus primers (HPVpU-1M: 5′-TGTCAAAAACCGTTGTGTCC-3′ and HPVpU-2R: 5′-GAGCTGTCGCTTAATTGCTC-3′), using the TaKaRa PCR Human Papillomavirus Typing Set (Takara Bio Inc.)^33^ at the E6 and E7 homologous regions of HPV (228–268 bp). When the HPV genotype could not be determined via this procedure, the region containing the HPV L1 gene was amplified using the primer set GP5+/GP6+ (GP5+: 5′-TTTGTTACTGTGGTAGATACTAC-3′ and GP6+: 5′-GAAAAATAAACTGTAAATCATATTC-3′). PCR products were purified using a PCR Clean-up kit (Takara Bio Inc.). Sanger sequencing was performed using the ABI 3130xl DNA Sequencer (Applied Biosystems) according to the manufacturer’s instructions. HPV genotypes were determined when the concordance rate between the obtained sequences and the HPV genotypes registered in the GenBank database was over 95% using BLAST (https://blast.ncbi.nlm.nih.gov/Blast.cgi). For patients whose HPV genotypes could not be determined via Sanger sequencing, we performed HPV in situ hybridization (ISH) using HPV-III High-Risk probes (Roche Diagnostics, Mannheim, Germany) to confirm HPV infection. This assay detected high-risk HPV genotypes, including HPV 16, 18, 31, 33, 35, 45, 52, 56, 58, and 66. Patients were diagnosed to be HPV-negative when HPV DNA could not be detected via HPV-ISH.

### Evaluation of p16 via immunohistochemistry (IHC)

IHC for p16 was performed in deparaffinized 4 μm FFPE sections. p16 IHC is widely used to facilitate the diagnosis of HPV. A p16 antibody (1:50, p16^INK4a^, G175-405; BD Biosciences, San Jose, CA, USA) was used to perform IHC for p16^34^. Each section was exposed to 0.3% hydrogen peroxide for 15 min to block endogenous peroxidase activity. For staining, an automated stainer (Dako, Carpinteria, CA, USA) was used according to the manufacturer’s protocol. The ChemMate EnVision method (Dako) was used for detection. Appropriate positive and negative controls were used for each antibody, and only tumors with a positive p16 expression both in the nucleus and cytoplasm were classified as p16-positive (Supplementary Fig. S1).

### Detection of PD-L1 expression using IHC

PD-L1 expression in tumor specimens of patients with ASCC was assessed using the commercially available PD-L1 IHC 22C3 pharmDx assay (Dako) (Agilent Technology, Santa Clara, CA, USA), which is linked to the use of pembrolizumab^35^. Positive PD-L1 expression in ≥ 1% of all tumor cells was set as the TPS and classified as positive, consistent with the methodology used in other studies involving anti-PD-1 antibody treatment of multiple cancers (Supplementary Fig. S2)^36^.

### Deep sequencing for hotspots in 50 cancer-related genes

Genomic DNA was quantified using the Qubit dsDNA HS Assay Kit (Thermo Fisher Scientific, Waltham, MA, USA) according to the manufacturer’s protocol, and 50 ng of genomic DNA was used to create the libraries with Ion AmpliSeq Cancer Hotspot Panel v2 (Thermo Fisher Scientific) that targets approximately 2800 COSMIC mutational hotspot regions of 50 cancer-related genes and was standardized for clinical molecular testing across the entire institution. The libraries were quantified using real-time RT-PCR (TaqMan). Sequencing was performed using an Ion Proton platform (Thermo Fisher Scientific). For quality control, samples with a mean read depth of coverage of over 1000 and a base quality score of 20 (≤ 1% probability of being incorrect) were selected, which accounted for 90% of the total reads.

### Classification of oncogenic/pathogenic mutations in 50 cancer-related genes

Sequencing reads were mapped to the UCSC human reference genome (GRCh37), and data analysis was carried out using the Torrent Suite Software v5.0.4 (Thermo Fisher Scientific). First, somatic mutations were selected as follows: (1) The variant allele frequency of somatic mutations of more than 4% in tumor tissues was included; (2) single nucleotide polymorphisms were removed if they showed a threshold allele frequency of ≥ 0.01 in either the NHLBI GO Exome Sequencing Project (ESP6500) (http://evs.gs.washington.edu/EVS/) or the Integrative Japanese Genome Variation Database (iJGVD, 20181105) (https://ijgvd.megabank.tohoku.ac.jp/)^37^; (3) the mutations were registered as “pathogenic/likely pathogenic variants” in ClinVar^38^ or as “oncogenic/likely oncogenic variants” in the OncoKB dataset (http://oncokb.org) (using oncokb-annotator ver 2.2.0, https://github.com/oncokb/oncokb-annotator/tree/v2.2.0) were included^7^. Finally, all the selected variants were manually checked using the Integrative Genomics Viewer (http://www.broadinstitute.org/igv/)^39^.

### Distribution of somatic ASCC mutations in the database

Somatic mutations obtained from targeted sequencing data from the American Association for Cancer Research (AACR) Genomics Evidence Neoplasia Information Exchange (GENIE) project were downloaded as a mutation annotation format file via the Synapse Platform (http://www.synapse.org/genie) (GENIE version 8.1-public). Somatic mutations were filtered using the following criteria: (1) Single nucleotide polymorphisms were removed if they showed a threshold allele frequency of ≥ 0.01 in either the NHLBI GO Exome Sequencing Project (ESP6500) (http://evs.gs.washington.edu/EVS/) or the Integrative Japanese Genome Variation Database (iJGVD, 20181105) (https://ijgvd.megabank.tohoku.ac.jp/); (2) mutations were registered as “pathogenic/likely pathogenic variants” in ClinVar or as “oncogenic/likely oncogenic variants” in OncoKB dataset^7^ (http://oncokb.org) (using oncokb-annotator ver 2.2.0, https://github.com/oncokb/oncokb-annotator/tree/v2.2.0) or as “high-impact variants” in SnpEff ver 4.3 using GRCh37.75 databases (http://snpeff.sourceforge.net). Within high-impact variants, nonsense mutations, such as frameshift, stop-gain, stop-loss, and start-loss mutations, were included, and other variants were excluded. Finally, among these filtered variants, recurrent variants in addition to variants observed at a frequency of 5% or more were validated. Somatic mutations and copy number alterations were categorized into four levels of evidence. Gene aberrations with evidence levels 1–3A were judged as actionable mutations for molecularly targeted drugs^7^.

### Statistical analysis

The association between HPV status and clinicopathological factors was examined using Fisher’s exact test. Continuous variables were compared using the Mann–Whitney U test. All *p* values were two-tailed, and *p* values < 0.05, were considered statistically significant. As for the efficacy of CCRT, we evaluated the response rate using the Response Evaluation Criteria in Solid Tumors guidelines (RECIST version 1.0). PFS was estimated by the Kaplan–Meier method and defined as the interval from the initial date of CCRT to the date of progression or death from any cause. We compared PFS depending on p16 expression using the log-rank test. Statistical analysis was performed using R software ver. 3.6.0 (R Foundation, Vienna, Austria) and the SPSS (version 27.0, IBM, Armonk, NY, USA).

## Supplementary Information

Supplementary Information 1.

Supplementary Information 2.

## Data Availability

All data generated or analyzed during this study are included in this manuscript or in the Supplementary Materials.

## Abbreviations

AACR: American Association for Cancer Research
ASCC: Anal squamous cell carcinoma
CCRT: Concurrent chemoradiation therapy
CR: Complete response
NCCH: National Cancer Center Hospital
FFPE: Formalin-fixed paraffin-embedded
GENIE: Genomics Evidence Neoplasia Information Exchange
HIV: Human immunodeficiency virus
JSCCR: Japanese Society for Cancer of the Colon and Rectum
PFS: Progression-free survival
RECIST: Response evaluation criteria in solid tumors
TPS: Tumor proportion score
