# Supplementary material for: Comparison of clinicopathological and genomic profiles in anal squamous cell carcinoma between Japanese and Caucasian cohorts
Source: Sci Rep. 2023 Mar 3;13:3587. doi: 10.1038/s41598-023-30624-w (PMC9984524; doi:10.1038/s41598-023-30624-w)
Supplement: Supplementary file 1 — Supplementary Information 1. [file 41598_2023_30624_MOESM1_ESM.pdf]

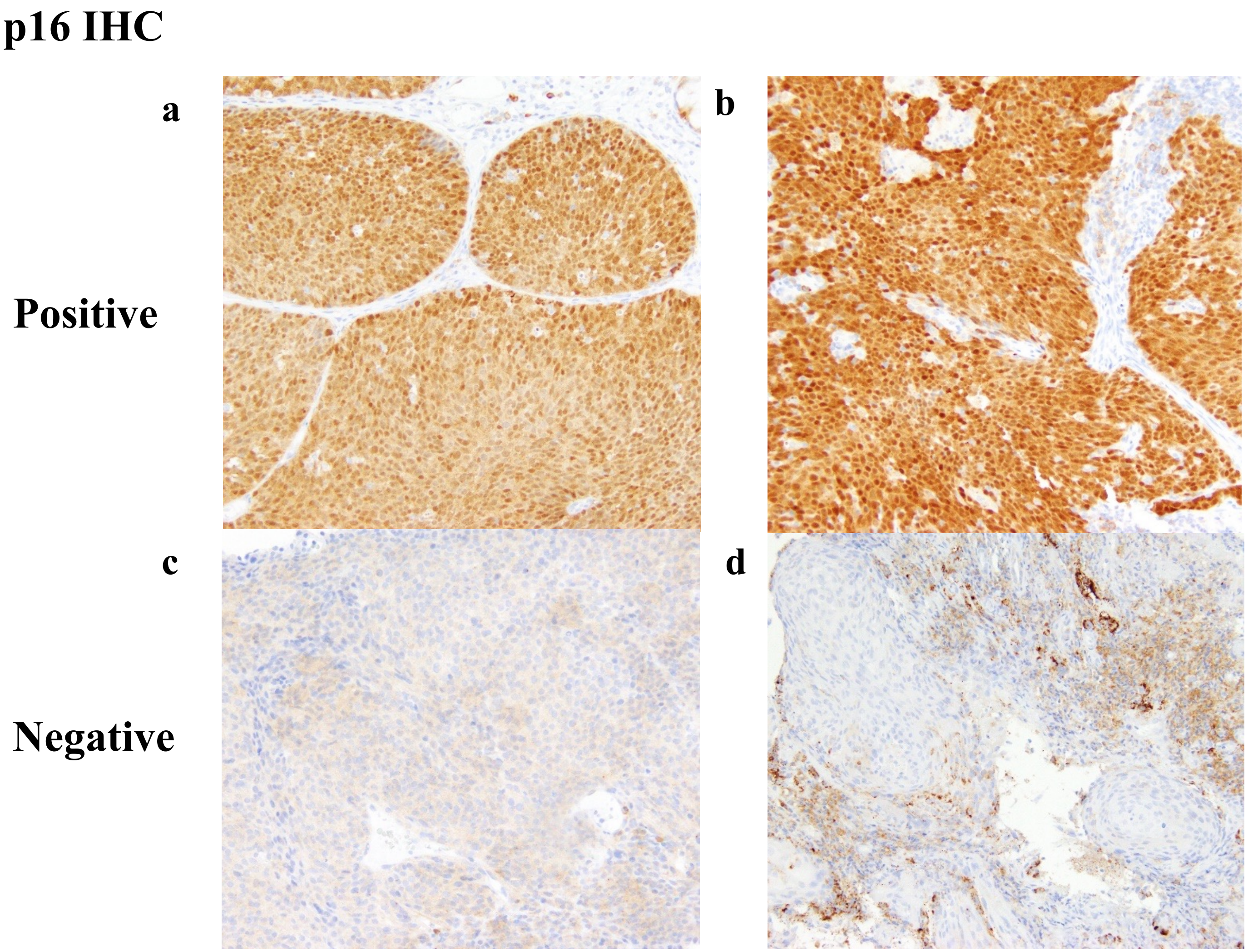

Supplementary Fig. S1 Immunohistochemical detection of p16 expression in 41 clinical specimens. (a, b) The above two images show p16-positive cases. (c, d) The below two images show p16-negative cases.

a b

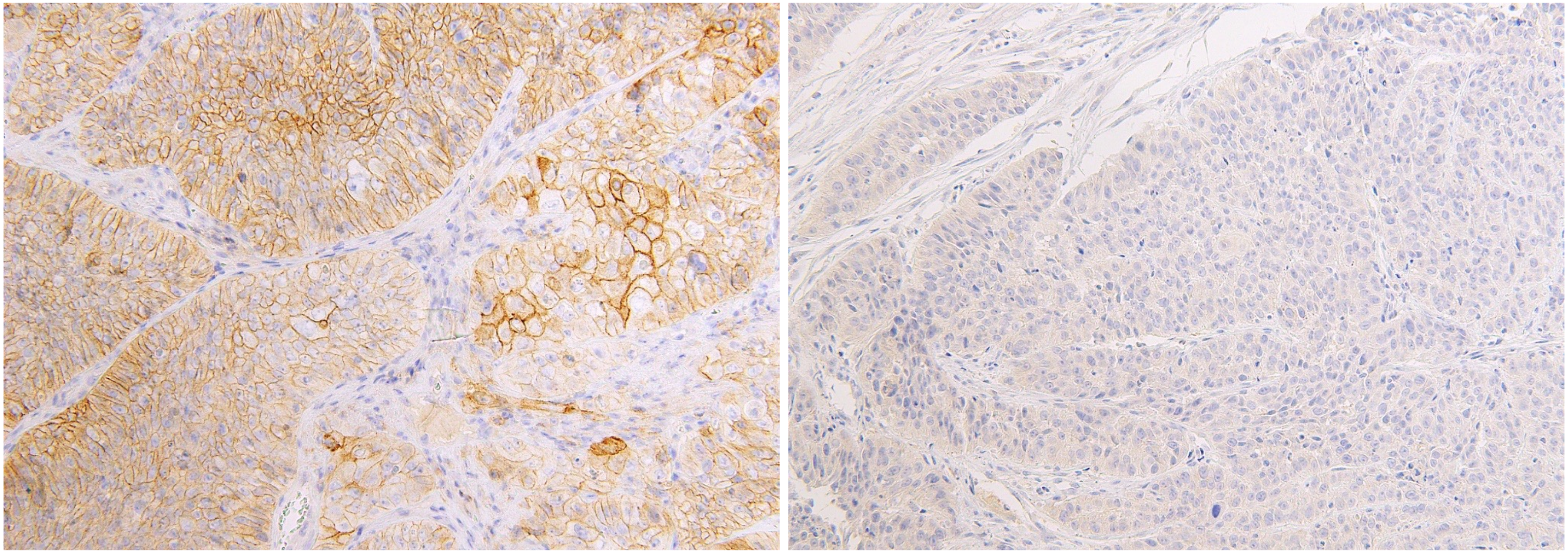

Supplementary Fig. S2 Immunohistochemical detection of PD-L1 expression in 41 clinical specimens. (a) This image shows an example of a PD-L1-positive sample. (b) This image shows an example of a PD-L1-negative sample.

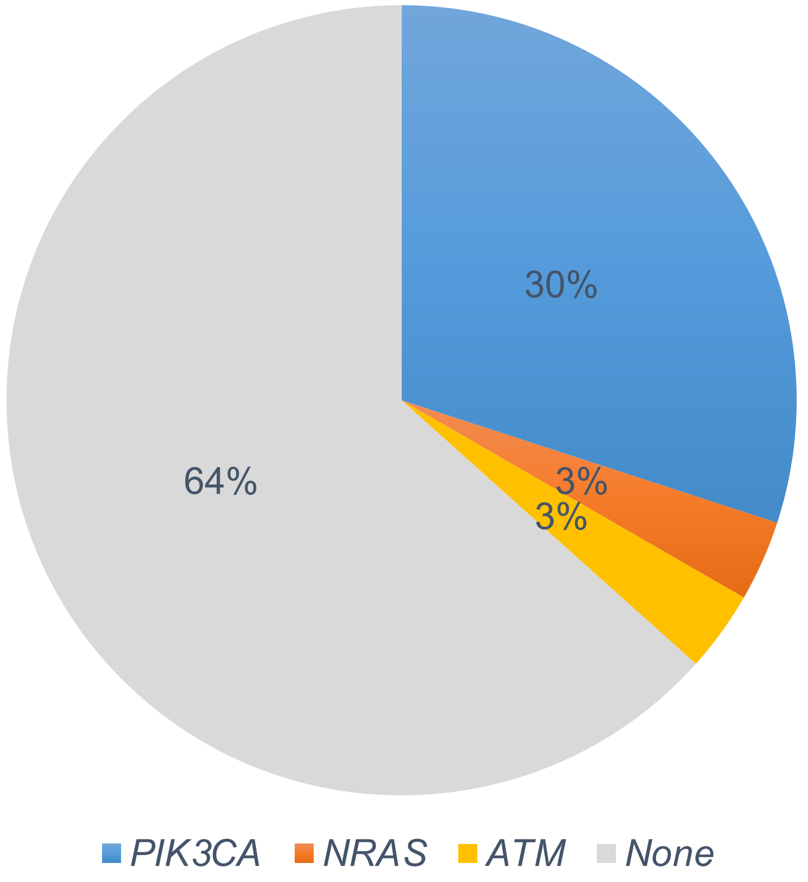

Supplementary Fig. S3 The fraction of actionable genomic alterations in our cohort.

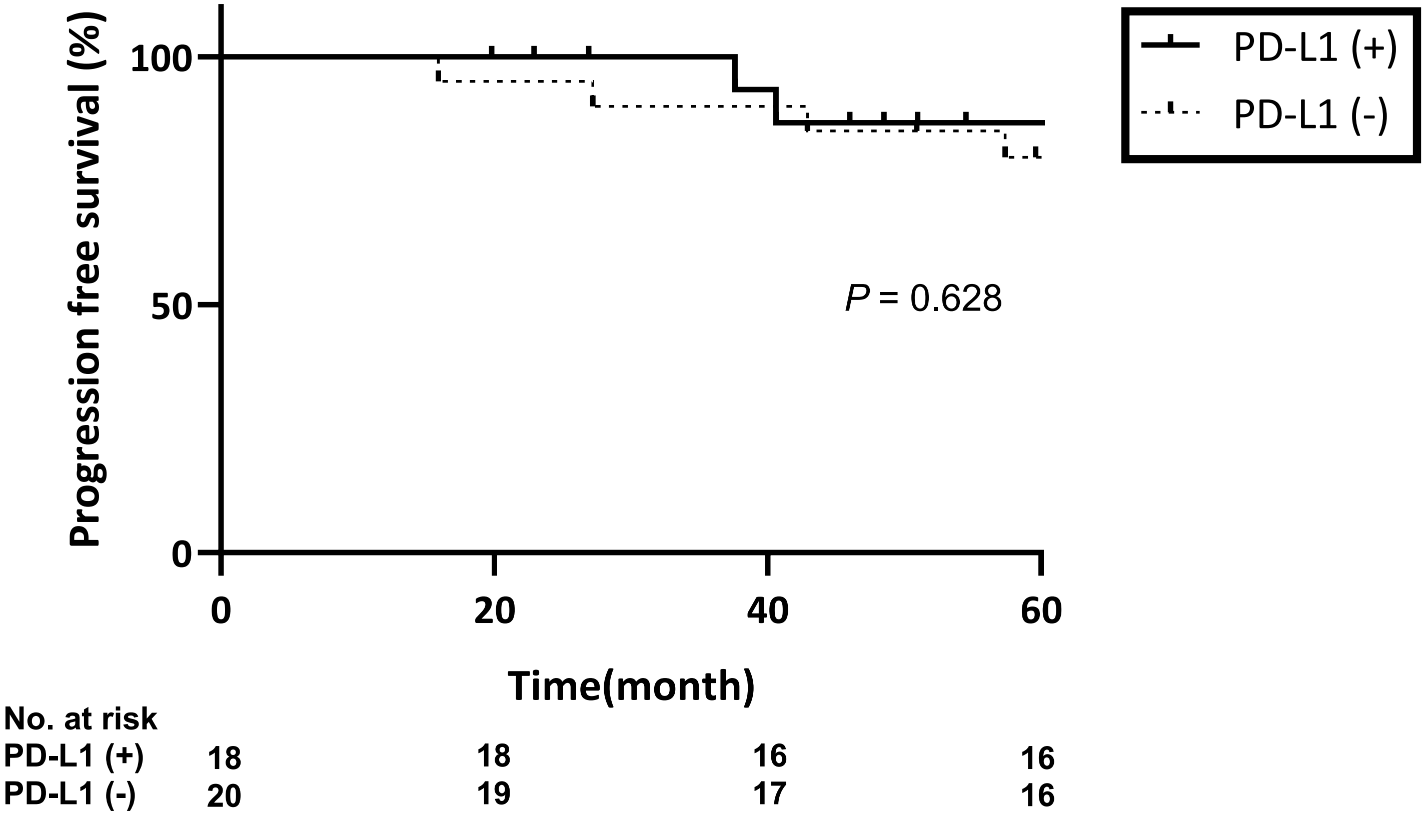

Supplementary Fig. S4 Kaplan-Meier analysis of PD-L1 expression and progression-free survival in 38 patients receiving CCRT. Patients with PD-L1 expression in ≥ 1% of all tumor cells are represented with a straight line and those without PD-L1 expression with a dashed line.
